# Media Exposure to Climate Change, Anxiety, and Efficacy Beliefs in a Sample of Italian University Students

**DOI:** 10.3390/ijerph18179358

**Published:** 2021-09-04

**Authors:** Daniela Acquadro Maran, Tatiana Begotti

**Affiliations:** Department of Psychology, Università di Torino, Via Verdi 10, 10124 Torino, Italy; tatiana.begotti@unito.it

**Keywords:** climate anxiety, media exposure, self-efficacy

## Abstract

The climate crisis poses a serious threat to the health and well-being of individuals. For many, climate change knowledge is derived from indirect exposure to information transmitted through the media. Such content can elicit a variety of emotional responses, including anger, sadness, despair, fear, and guilt. Worry and anxiety are especially common responses, usually referred to as “climate anxiety”. The main objectives of this study were to analyze how exposure to climate change through the media relates to climate anxiety and individual and collective self-efficacy, and to evaluate the relationship between climate anxiety and efficacy beliefs. A total of 312 Italian university students (aged 18–26 years) participated in the research by filling out an anonymous questionnaire. Participants reported being exposed several times per week to information about climate change, especially from social media, newspapers, and television programs. Moreover, the results showed that the attention paid to information about climate change was not only positively related to climate anxiety, but also to individual and collective self-efficacy. Most notably, participants’ efficacy beliefs were found to be positively related to climate anxiety. This somewhat controversial finding stresses that, in the context of pro-environmental behavior changes, a moderate level of anxiety could engender feelings of virtue, encouraging people to rethink actions with negative ecological impacts.

## 1. Introduction

The climate crisis poses a serious threat to the health and well-being of individuals, to which societies must urgently respond and adapt [1]. Multiple studies have investigated the effects of climate change on health [2,3,4,5]. These effects include an increase in mortality rates and injuries resulting from natural disasters and other extreme weather events, and an increased risk of malnutrition, physical illness, and psychological distress [6,7,8]. The relationship between acute meteorological disasters, such as floods, heat waves, and cyclones, and the subsequent anxiety experienced, is particularly well-documented [9,10,11,12], evidencing the threat of climate change to the mental health of individuals. Ogunbode et al. [13] showed that mental health and psycho-social well-being can be influenced both directly, for example, through life experiences related to extreme weather events or natural disasters, and indirectly, through exposure to climate change news. Both have been found to produce a general increase in mental illness and emotional difficulties. Similarly, Fritze et al. [1] argued that exposure to extreme weather events, including prolonged indirect exposure to, and awareness of, the effects, have substantial repercussions in terms of both morbidity and psychological disorders. These repercussions can be either moderated or exacerbated according to a variety of psycho-social factors [14], including individual and community-wide vulnerabilities [15,16,17]. In their review of the relevant literature, Doherty and Clayton [18] identified, among the effects of indirect climate change exposure, intense emotional arousal, including anxiety around both current and future risks to humans and other species.

For many, climate change knowledge is derived from indirect exposure to information transmitted through the media and social media networks. Such content can elicit a variety of emotional responses, predominantly negative, including anger, sadness, despair, fear, and guilt. Worry and anxiety are especially common responses. This phenomenon is commonly referred to as “eco-anxiety” or “climate anxiety,” and is associated with symptoms such as panic attacks, loss of appetite, irritability, weakness, and sleep disturbance [8]. From this perspective, climate change may be seen as a threat to the mental health of populations on a potentially global scale. 

### 1.1. Media Exposure and Climate Anxiety

The terms “climate anxiety” and “eco-anxiety” are used to describe the psychological discomfort that results from knowledge and/or experience of environmental degradation and the associated risks [19,20]. Maibach et al. [21] found that 18% of their sample of 2129 U.S. citizens felt alarmed by climate change and were experiencing feelings such as sadness, anger, and fear. According to an AXA-IPSOS survey that involved 13,000 people from three continents [22], one-third of the respondents said that climate change could have an impact on their personal well-being. Moreover, an Italian survey conducted in 2019 involving a representative national sample of 800 young adults indicated that, for 51% of them, climate change represents the primary source of their distress [23]. In recent years, discourse on climate change has described it as “catastrophic,” “rapid,” “urgent,” “irreversible”, and “chaotic” [24]. Such language is characteristic of a new way of narrating the climate crisis that Risbey [17] terms “alarming” [25,26]. However, according to Vu, Liu, and Tran [27], media coverage of climate change (particularly given the media outlets’ political and/or ideological agendas) is a controversial topic, and many media outlets are criticized for inadequately covering the climate crisis or for not doing enough to highlight its severity (see University of Kansas [28]). Politicians and other influential public figures have contributed to this new narrative (either by over- or underestimating the crisis), as have media and social media networks [29,30,31,32,33]. Investigations by Stamm et al.. [34], Carvalho and Burgess [29], and Olausson [35] have demonstrated the agenda-setting role of the media. Consisting of the creators and distributors of images and narratives circulated among the public, the media is largely responsible for the transmission of scientific knowledge and its incorporation into daily experiences [35]. Through the application of standard principles and procedures, the media determines which events will become “news”, presenting interpretations of an event’s causes and consequences in accordance with their own ideological and political purposes and preferences [29]. A study by Brulle, Carmichael, and Jenkins [36] identified various factors responsible for public anxiety in the United States between 2002 and 2010. Using data from 74 different surveys conducted over nine years, the authors highlighted media coverage as among the primary factors influencing the extent to which participants experienced anxiety around climate change. Climate change information in the media seems to have a significant impact on levels of public anxiety. A positive correlation was found between the two variables [37].

### 1.2. Media Exposure, Self-Efficacy, and Collective Efficacy

Albrecht [38] identified the perceived inability of individuals to respond to environmental challenges as a form of anxiety termed “ecoparalysis”. Moser and Dilling [39] and Lorenzoni et al. [40], identified various barriers to modifying attitudes and behaviors at two interrelated levels: individual and social. The climate change issue acts as a global narrative that connects and contextualizes multiple events on a global scale, influencing judgments about responsibilities and the effectiveness of interventions. The data analyzed by Lorenzoni et al. [40] were taken from three studies of the U.K. population. These studies, although not originally designed for this purpose, identified the key obstacles and barriers to pro-environmental action: lack of knowledge, uncertainty and skepticism, the outsourcing of responsibilities, apathy [41], and the mistrust of information sources. The outsourcing of responsibilities and skepticism concerning the reality of climate change and the effectiveness of mitigation efforts may be coping mechanisms that moderate negative emotional responses, including anxiety, through denial.

Moreover, the media coverage of climate change’s impacts and responses tends to be saturated with threat information undermining perceived self-efficacy [42]. A study by Kellstedt et al. [43] found that individuals who reported high exposure to information about global warming also reported feeling less able to effectively address the issue. Hibberd and Nguyen [44] reported that young people (aged 18–26 years) have a strong sense of behavioral inefficacy about climate change that is impacted by messages in the media and social media networks. Therefore, there is a growing body of research evidencing that exposure to information about climate change through sources such as these can reduce efficacy beliefs and increase climate anxiety [45]. 

### 1.3. Climate Anxiety, Self-Efficacy, and Collective Efficacy

The relationship between climate change, anxiety, and self-efficacy is a subject of ongoing debate. Nabi et al. [46] claimed that self-efficacy could be affected by the negative emotions evoked through exposure to climate change information. According to Lowe et al. [47], “research has shown that people feel overwhelmed by shocking images and, although it heightens their concern, it also reduces their self-efficacy to take action and lessen these events through personal action” (p. 451). However, other studies show that individuals experiencing a certain degree of climate anxiety tend to experience higher levels of self-efficacy [48,49,50]. This response may represent a form of mitigation adopted by individuals who feel more capable of intervening to reduce the impacts of environmental degradation or, at least, of minimizing the damage to the environment [51]. Collective self-efficacy is the belief that a group, or groups, can successfully perform a specific task [52,53,54]. In the context of climate change, collective efficacy focuses on the effectiveness of group mitigative action, the impact on future generations, and a sense of collective responsibility for the health of the planet [55]. A sense of collective self-efficacy may therefore decrease climate anxiety by restoring the confidence of individuals in the belief that, working together, they can effectively address the climate crisis [56,57,58,59].

The primary objective of this study was to evaluate the effects of media exposure to climate change information in a sample of university students in terms of attention paid and the source type. The second aim of this study was to analyze how exposure to climate change through the media relates to climate anxiety and perceived individual and collective self-efficacy. On the basis of the literature referenced above, we hypothesized a positive correlation between attention paid to climate change information and climate anxiety (the greater the attention paid, the more anxiety one experiences), and a negative correlation between climate change information exposure and self-efficacy beliefs (the greater the exposure, the less individual and collective self-efficacy one experiences). The third and final aim of this study was to analyze the relationship between climate anxiety and efficacy beliefs. Given the aforementioned inconsistencies between the results of previous studies, we proceeded without a specific hypothesis, instead adopting an exploratory perspective. 

We chose to limit the scope of the investigation to young adults aged 18–28 years. This choice was made in accordance with Wu et al.’s [60] findings indicating that young people are more prone than adults to the psychological, emotional, and physical effects of climate anxiety given their development stage and the consequent vulnerability to anxiety. 

This study will help clarify the somewhat controversial relationship between climate change information exposure, efficacy beliefs and climate anxiety.

## 2. Methods

### 2.1. Participants

A total of 312 students enrolled in undergraduate and postgraduate humanities courses at the University of Turin participated in the research (25% men and 74% women; 1% of the sample opted not to specify their gender). Their ages ranged from 18 to 28 years (mean = 21.58; SD = 1.96). Participants took part in the research on a voluntary basis, and no compensation (or extra credit) for their participation was provided.

### 2.2. Measures

The data were collected with an anonymous questionnaire. The items and data are from the MECAMH project (English version of the questionnaire available at https://osf.io/6n4rb/?view_only=d708efc61e6945a1bc02e037a3ccac96 (accessed on 30 October 2019); [61]).

Media exposure to climate change information was investigated in relation to the following variables:

(a). Attention paid to information about climate change, measured through one item; possible responses were: none (coded as 0), a little (coded as 1), some (coded as 2), and a lot (coded as 3).
(b). Information source: the frequency of reading or listening to climate change information from different sources measured through 9 items, one for each source (such as TV, YouTube, books, friends etc.). Possible responses ranged from never (coded as 0) to more than 10 times per day (coded as 8).

Climate anxiety was investigated through a scale modelled on the State-Trait Anxiety Inventory (STAI Y1) [62,63]. It included 7 items assessing the participants’ present feelings around climate change. The feelings included in the scale were: calm (reverse-coded), tense, relaxed (reverse-coded), anxious, peaceful (reverse-coded), worried, and terrified. Possible responses ranged from not at all (coded as 1), to somewhat (coded as 2), moderately (coded as 3), very much (coded as 4), and extremely (coded as 5). The range of the scale was from 7–35. In this study, the Cronbach’s alpha for the scale was 0.89.

Climate self-efficacy (individual and collective) was investigated through the Perceived Climate Self-Efficacy Scale [64,65,66]. It includes 5 items pertaining to individual self-efficacy, and 5 items pertaining to collective self-efficacy. Participants were asked to express the extent to which they agreed with a variety of statements. For example, “I trust that I can do my part to solve the climate crisis” or “I trust that we, as young people, can contribute to solving the climate crisis”. Possible responses ranged from strongly disagree (coded as 1), to disagree (coded as 2), neither disagree nor agree (coded as 3), agree (coded as 4), and strongly agree (coded as 5). The range of each scale was from 5–25. In this study, the Cronbach’s alpha for individual climate self-efficacy was 0.73; the Cronbach’s alpha for collective climate self-efficacy was 0.79.

Statistical analyses were performed with IBM SPSS (IBM Corp. Armonk, NY, USA), release 25. Descriptive measures (means ± SD) were calculated for all test variables. Correlations were calculated to examine the relations between attention paid to information about climate change, climate anxiety, and individual and collective climate self-efficacy.

### 2.3. Procedure

The local ethical committee of the University of Turin approved this research project (protocol code 437036). The data were collected during the 2019/2020 academic year by research assistants specifically trained by the researchers. In accordance with the Declaration of Helsinki [67], both an information letter and an informed consent form were distributed among the participants alongside the questionnaire. The participants completed the questionnaire in their classroom at the university before the beginning of a lesson and returned it immediately (response rate 98%). Completing the questionnaire required approximately 20 min. 

## 3. Results

### 3.1. Descriptive Statistics

Most participants stated they paid either some or a lot of attention to information about climate change (89%), while only 11% stated they paid little or no attention. 

For each of the nine items measuring the frequency with which the participants read or listened to climate information from various sources, a mean of the scores was calculated (range 0: never to 8: more than 10 times per day). For results, see Table 1 (in decreasing order). 

As shown in Table 1, the top three sources of information were a social media network (Facebook), newspapers, and television programs. In Table 2, the results indicate that, on average, collective self-efficacy had a higher score than individual self-efficacy.

Table 2 details the descriptive statistics on climate anxiety and individual and collective self-efficacy.

### 3.2. Correlation Analysis

Table 3 shows the correlation analysis between the variables. With regard to media exposure, attention paid to information about climate change was considered. As evidenced, attention paid to information about climate change was positively related to climate anxiety, and both individual and collective self-efficacy. Moreover, climate anxiety was positively related to individual and collective self-efficacy.

## 4. Discussion

The present study sought to assess the effects of exposure to climate change information in the media in a sample of 18–26-year-old Italian university students. In particular, the relationship between climate change media information exposure, climate anxiety, and individual and collective self-efficacy was studied. The first interesting result of the investigation concerned media and social media network exposure. Participants reported being exposed several times per week to information about climate change. The results underlined that attention paid to information is positively related to climate anxiety. These findings confirm those of earlier investigations by Risbey [24], Olausson [35], Brulle et al. [36], and Marlon et al. [37], highlighting that media exposure is one of the main factors influencing climate anxiety and vice versa. 

Concerning the positive relationship between media exposure and individual and collective self-efficacy, the results show that when the attention paid to climate change increases, there is an increase in efficacy beliefs. These results may have been impacted by the information content. For example, information about how one might intervene as an individual, or mitigate best practices adopted by governments, may provide examples of pro-environmental behavior increasing efficacy beliefs and decreasing anxiety [68]. Moreover, as suggested by Moser [69,70,71], in communication about climate change, it is important to underline the reasons for those changes to improve people’s knowledge about the phenomenon and how to cope with it. Future research could consider how the content of the information to which participants are exposed influences its effects on climate anxiety, individual self-efficacy, and collective self-efficacy.

The findings regarding individual and collective self-efficacy are somewhat controversial. Correlational analysis revealed a positive relationship between individual and collective self-efficacy and climate anxiety, suggesting that an increase in self-efficacy (individual and collective) corresponds to an increase in anxiety, and vice versa. Thus, the levels of climate anxiety in our sample were not affected by eco-paralysis, as defined by Albrecht [38]. Moreover, our investigation demonstrated that participants experienced increased self-efficacy in response to opportunities to mitigate climate change, both as individuals and as a part of a group, even if they were experiencing climate anxiety. Interestingly, this result confirms those of Homburg and Solberg [48], Verplanken and Roy [72], Howell et al. [49], and Helm et al. [50]. In accordance with Pihkala [20], the results show that anxiety can play a positive role as “an emotion which engenders information-seeking and problem-solving” (p. 12). Kurth [73] termed this “practical anxiety” and, in the context of pro-environmental behavior changes, it provokes ethical and practical deliberation about what kind of behavior is needed to encourage people to rethink actions with negative ecological impacts. This “practical” climate anxiety can, for example, motivate individuals to make more sustainable lifestyle choices and increase their pro-environmental activity (for example, involving the neighborhood in shared gardens). Further research is needed to clarify the relationship between climate anxiety, individual and collective self-efficacy, and the willingness to undertake pro-environmental behaviors. Closer study of the behaviors enacted may prove useful as a means of measuring and better understanding how climate anxiety influences people’s willingness to engage in mitigative climate action (see, for example, Lorenzoni et al. [40], and Hart and Feldman [42]). According to Bradley et al. [74], engaging in pro-environmental behaviors may be an attempt to cope with and alleviate climate anxiety and may, with further study, prove useful for moderating the associated distress. This investigation’s findings contribute to a better understanding of how mitigative climate actions may function as effective coping strategies. 

Nevertheless, this study is not without limitations. First, because of the difficulty, novelty, and interdisciplinarity of the subject matter, not all relevant sources could be engaged within the article. Moreover, the research is cross-sectional and, therefore, the direction of associations and causality between constructs could not be established. Additionally, all participants were university students with a high level of education, potentially impacting their ability to evaluate the information sources and moderate their responses accordingly. Furthermore, the sample was not sex-balanced; it was composed primarily of women. In the meta-analysis by Su et al. [75], it emerged that women are more prone to experience higher levels of social media addiction than men. Therefore, the extent to which these findings apply to both men and women cannot be conclusively determined. The investigation did not take into consideration socio-demographic variables, such as family situation, income stability, or parental support. Specifically, the age and social group sampled may (more likely than others) display increased anxiety levels for different reasons, be it financial insecurity, class performance, the novelty of the university environment, and countless others. Anxieties may be transferred to a readily available explanation, climatic in this case, without deeper substantiation. Future investigation and statistical control for such confounding factors would be welcomed. Moreover, these variables may provide further insight into the participants’ perceived self-efficacy and/or collective self-efficacy in relation to climate change mitigation. For example, Darawshy et al. [76,77], in their investigation into the psychological consequences of community violence, found that collective self-efficacy moderated the relationship between witnessing community violence and the symptoms of post-traumatic stress. Further research could include variations in the participants’ levels of education and other socio-demographic characteristics. Social-desirability bias [78], that is, the participants’ tendency to provide responses they believe present a favorable image of themselves, may also have impacted the study’s findings. The divisive nature of this hotly debated topic, evident in the media, but also within family and friendship groups, arguably increases the likelihood of this bias, as does the topic’s overtly ethical dimensions. As such, it is difficult to determine the extent to which participants’ responses reflect their desire to appear concerned because they believe this is expected of them. With this in mind, future research would benefit from tools intended to assess and account for social-desirability bias in self-reporting [79]. Finally, the nature and extent of the participants’ climate anxiety were not evaluated and warrants further investigation. Verplanken and Roy [72] find worry about the environment to be a constructive form of concern. They distinguish between “habitual ecological worrying and pathological worry”, evidencing a near-zero correlation between the two. In their study of participants from Europe and the United States (*n* = 132), they identified a significant correlation between ecological worrying, environmentally conscious attitudes and behaviors, and the perceived opportunity for pro-environmental intervention. Similarly, the results of this study support show that some people who self-report climate anxiety also report efficacy beliefs. Nevertheless, according to Verplanken et al. [80], further investigation might clarify whether ecological worry is a precursor to climate anxiety on the spectrum of concern, and if there is a point at which constructive habitual ecological worry becomes destructive, pathological worry. 

## 5. Conclusions

In conclusion, this investigation demonstrates that, in line with previous research, the exposure of young adults to climate change information in the media and through social media networks is related to negative emotions, including anxiety, and to increased individual and collective self-efficacy beliefs about climate change. Most notably, the participants’ efficacy beliefs were found to be positively related to climate anxiety. It should be noted that the anxiety level of our sample was quite low. For this reason, it would be interesting to investigate this same relationship in a sample of subjects experiencing more severe climate anxiety (perhaps related to the direct experience of an adverse climate event) and evaluate the effects on self-efficacy in relation to this variable, and whether self-efficacy could play a different role contributing to the mitigation of anxiety. Since the literature provides different measures for climate anxiety, it could be interesting to make a comparison between them to better understand how to investigate the construct of climate anxiety (for example [81]).

Climate anxiety could be mitigated by constructive and accurate climate change narratives that underline how to cope with it [10]. The risk of overexposure and the existence of excessive climate disaster content could result in eco-paralysis, as described by Albrecht [38]. There is the possibility of inaction relating to inaccurate information about risk exposure. Media coverage of best practices in pro-environmental behavior (both at the individual and societal level) may mitigate this unfavorable result, alongside published data about differentiated waste collection, new inventions, and policy and structural reforms that could help to improve the efficiency of electrical appliances, cars, and other objects of daily use [69]. The dissemination of news may encourage the uptake of pro-environmental behaviors and begin a virtuous cycle in line with Verplanken and Roy’s [72] account of the “usual worry” about climate change coinciding with positive ecological attitudes and behaviors and modeling a positive example for others to follow. This is particularly important for young people whose lives are already being impacted by climate anxiety, and who, without urgent mitigative action, will see the effects of climate change worsen considerably during their lifetimes.

## Figures and Tables

**Table 1:** Source of Information about climate change (*n* = 312).

	*M*	SD
Facebook	4.62	2412
Printed and online newspapers	4.03	1984
TV news and other TV programs	3.94	1927
Family, friends and colleagues	3.87	1791
Scientific articles/journals/blogs	3.02	2362
Radio news and radio programs	2.33	1948
YouTube	2.31	2364
Books and magazines	2.16	2102
Twitter	1.59	2615

Note: *M* = mean; SD = standard deviation.

**Table 2:** Descriptive statistics (*n* = 312).

	*M*	SD	Range
Climate anxiety	23.85	5.13	7–35
Individual self-efficacy	17.42	2.92	5–25
Collective self-efficacy	19.23	2.93	5–25

Note: *M* = mean; SD = standard deviation; Range = lowest and highest values of the scale.

**Table 3:** Correlation analysis.

	AI	CA	ISE	CSE
AI	-			
CA	0.364 **	-		
ISE	0.291 **	0.304 **	-	
CSE	0.278 **	0.211 **	0.675 **	-

** Correlation is significant at 0.01. * Correlation is significant at 0.05. Note: AI = attention paid to information about climate change; CA = climate anxiety; ISE = individual self-efficacy; CSE = collective self-efficacy.

## Data Availability

The datasets generated for this study are available on request to the corresponding author.
